# MicroRNA-155 governs SHIP-1 expression and localization in NK cells and regulates subsequent infiltration into murine AT3 mammary carcinoma

**DOI:** 10.1371/journal.pone.0225820

**Published:** 2020-02-10

**Authors:** Wendy M. Kandell, Sarah S. Donatelli, Thu Le Trinh, Alexandra R. Calescibetta, Tina So, Nhan Tu, Danielle L. Gilvary, Xianghong Chen, Pingyan Cheng, William A. Adams, Yin-Kai Chen, Jinhong Liu, Julie Y. Djeu, Sheng Wei, Erika A. Eksioglu

**Affiliations:** 1 Department of Immunology, H. Lee Moffitt Cancer Center, Tampa, Florida, United States of America; 2 Cancer Biology Ph.D. Program, University of South Florida, Tampa, Florida, United States of America; 3 Dong Nai Technology University, Dong Nai Province, Vietnam; Istituto Superiore di Sanità, ITALY

## Abstract

NK cell migration and activation are crucial elements of tumor immune surveillance. In mammary carcinomas, the number and function of NK cells is diminished, despite being positively associated with clinical outcome. MicroRNA-155 (miR-155) has been shown to be an important regulator of NK cell activation through its interaction with SHIP-1 downstream of inhibitory NK receptor signaling, but has not been explored in regard to NK cell migration. Here, we explored the migratory potential and function of NK cells in subcutaneous AT3 in mice lacking miR-155. Without tumor, these bic/miR-155^-/-^ mice possess similar numbers of NK cells that exhibit comparable surface levels of cytotoxic receptors as NK cells from wild-type (WT) mice. Isolated miR-155^-/-^ NK cells also exhibit equivalent cytotoxicity towards tumor targets *in vitro* compared to isolated WT control NK cells, despite overexpression of known miR-155 gene targets. NK cells isolated from miR-155^-/-^ mice exhibit impaired F-actin polymerization and migratory capacity in Boyden-chamber assays in response chemokine (C-C motif) ligand 2 (CCL2). This migratory capacity could be normalized in the presence of SHIP-1 inhibitors. Of note, miR-155^-/-^ mice challenged with mammary carcinomas exhibited heightened tumor burden which correlated with a lower number of tumor-infiltrating NK1.1^+^ cells. Our results support a novel, physiological role for SHIP-1 in the control of NK cell tumor trafficking, and implicate miR-155 in the regulation of NK cell chemotaxis, in the context of mammary carcinoma. This may implicate dysfunctional NK cells in the lack of tumor clearance in mice.

## Introduction

Natural Killer (NK) cells are a subset of lymphocytes that produce pro-inflammatory cytokines such as IFNγ and perforin, and kill target cells through an array of germline encoded receptors. NK cell activation is a finely tuned balance between positive (activating) and negative (inhibitory) signals. Ligands for these activating receptors are found on malignant or virally infected cells, which also frequently downregulate MHC [1]. A robust NK cell response in cancer patients correlates with a positive prognosis [2, 3], and these clinical data translate to animal studies showing that NK cell depletion or inactivation increases tumor burden and worsens prognosis [4, 5]. This highlights the important role of NK cells in anti-tumoral defense. NK are found within tumor infiltrating lymphocytes (TIL), however they are often rendered dysfunctional by means of the tumor [6]. In the context of disease, NK cells quickly respond to chemokine signals such as that of the abundantly produced chemoattractant CCL2 [7–9] elicited by malignant cells or other inflammatory leukocytes, making them early-responders at the scene of a challenge. While previous studies have shown that CCL2 is required for NK cell-mediated clearance of viral infections [10], information about NK cell chemotaxis in the context of breast tumor challenge is limited compared to T cell trafficking in the disease, and NK trafficking in other tumor types such as colon [11].

One class of regulators involved in diverse cellular processes are microRNAs (miRs), a class of small noncoding RNAs that post-transcriptionally represses gene expression by binding to transcripts exhibiting sequence homology, and inducing transcript degradation or inhibiting translation [12]. Deficiency of Dicer, an RNAse required for functional miRNA maturation, leads to defective NK cell development, solidifying the importance of miRNA regulation within NK cells [13]. In particular, microRNA-155 (miR-155) is expressed in NK cells and other leukocytes [14, 15], where it is upregulated by inflammatory stimuli like Toll-like receptor ligands, IFNβ, TNFα and IFNγ [16], and is robustly induced in response to activating cytokines IL-12 and IL-18 [17]. Several genes have been identified as functional targets of miR-155, including SH2-containing inositol polyphosphate 5-phosphatase (SHIP-1) [18], which negatively regulates IFNγ production in NK cells [17, 19]. Additionally, SHIP-1 regulates the actin cytoskeleton at various levels by interacting with filamin-1, a scaffolding protein that organizes actin filaments in ruffle formation during chemotaxis [20, 21]. Illustrating this relationship, decreases in filamin-1 or SHIP-2, a SHIP-1-related inositol phosphatase, leads to reduced F-actin polymerization in response to endothelial growth factor stimulation [22]. Furthermore, SHIP-1 is involved in the regulation of migration of murine neutrophils in response to chemoattractive agents [23]. Taken together, these data support a role for SHIP-1 not only in the regulation of cytokine secretion, as shown by Trotta et. al. [17] but also cell motility.

MiR-155 is processed from the transcript of *bic*, a non-protein-coding gene [24]. To study the physiological function of *bic*/miR-155 in NK cells, we utilized the *bic*/miR-155-deficient mouse (miR-155^-/-^) [17, 24]. Previous work has elucidated roles of miR-155 in NK cells both in vitro and in vivo, but impairments in chemotaxis are relatively unexplored. These works have shown that miR-155 is important for NK cell biology in vivo, showing such defects as impaired Interferon Gamma production and homeostatic proliferation [17, 25, 26]. However NK cells from miR-155^-/-^ mouse spleen displayed normal frequencies, and displayed a similar percentage of CD94, a marker of activation [25]. MiR-155 has also been shown to play a significant role in actin polymerization through the activity of SHIP-1 [17]. Given that miR-155 has not been shown to affect NK cytotoxicity [25], we hypothesized that accelerated tumor growth in mice may be due to reduced migratory capacity secondary to defects in actin polymerization in NK cells. To examine this, we isolated NK cells from mice genetically lacking miR-155, and conducted a series of functional assays, as well as chemotaxis, transwell assays, and demonstrate that miR-155 may regulate chemotaxis and subsequent NK tumor infiltration downstream of SHIP-1 in mammary carcinoma.

## Results

### NK cells in bic/miR-155^-/-^ mice retain normal cytotoxic function

MiR-155 has myriad roles in NK cell functionality, and thus we examined the miR-155^-/-^ mouse to interrogate this population. NK cells in these miR-155^-/-^ mice displayed normal levels of cytotoxicity receptors and frequencies in the spleen when compared to wildtype, similar to what has been previously characterized [17, 25]. Our results were similar to the findings cited, whereby the percentage of splenic NK cells was equivalent in miR-155^-/-^ and WT mice, as assessed by NK1.1/DX5 co-expression [1] (S1A and S1B Fig), suggesting that miR-155 does not play a role in NK cell development or homeostasis. NK are typically activated in inflammatory conditions. We thus assessed the cytotoxic potential of these cells post-activation with IL-2. This cytokine was chosen as the standard for in vitro NK activation, though other gamma chain cytokines are sufficient. [27]. Splenic NK cells were purified by negative selection, activated with IL-2 (100 U/ml) for 72 h, and assessed for functional phenotype by means of their activating receptors by flow cytometric analysis and additionally, cytotoxicity towards tumor targets. To this end, chromium release assay was performed, and similar similar lytic function towards YAC-1 murine lymphoma targets, as well as C57BL/6 syngeneic AT3 murine mammary carcinoma targets were seen in both WT and miR-155^-/-^ derived cells (S1D Fig). We found no differences in cell surface expression that might impair cytolytic function, as miR-155^-/-^ NK cells did not show defects in expression of the activating receptors NKp46, NKG2D or 2B4 (S1C Fig). Despite possessing comparable direct lytic function and an intact activating receptor phenotype, other properties of NK cells equally important for effective rejection of cancer can be affected by miR-155 deficiency. To follow this further, were particularly interested in dysregulation of the previously identified miR-155 target, SHIP-1 [18], as SHIP phosphatase activity negatively regulates NK function.

### miR-155 is linked to SHIP1 and influences chemotaxis

**Fig 1:**
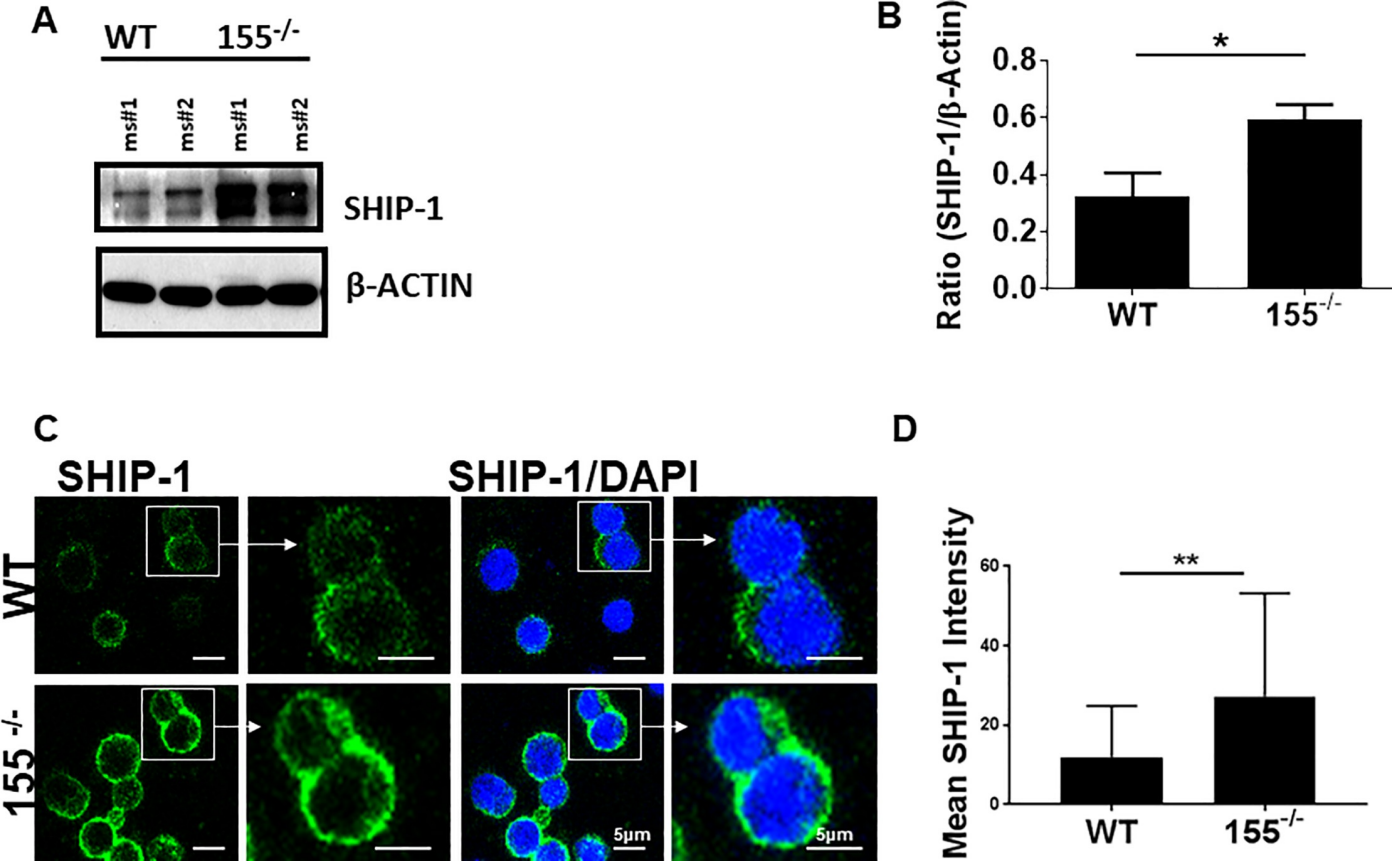
miR-155^-/-^ NK cells overexpress SHIP-1 and do not polymerize actin. IL-2 activated NK cells were generated from purified splenic wild-type or miR-155-/- NK cells. A) SHIP-1 immunoblot analysis of wild-type (lanes 1–2) or miR-155^-/-^ (lanes 3–4). IL-2 activated NK cells were derived from four mice per strain. B) Quantification of the mean ratio of SHIP-1 to actin from densitometry of immunoblots. C) IL-2 activated NK cells immunostained for SHIP-1. Data shown is a representative of three experiments performed unless otherwise stated. D) Quantification of mean SHIP-1 membrane intensity from confocal microscopy as in panel C.

Since SHIP-1 is a defined target of miR-155, and given the previous work on miR-155 regulation of SHIP-1 in NK cells [17, 25, 26], we assessed the level of total SHIP-1 in purified NK. Through this, we found that SHIP-1 was upregulated in purified NK cells of miR-155^-/-^ mice (Fig 1A and 1B**)**. We additionally performed immunofluorescence on NK cells isolated from WT or miR-155^-/-^ mice, and found increased cell-surface localization of SHIP-1 in cells lacking miR-155^-/-^ (Fig 1C and 1D**),** suggesting that not only is SHIP-1 upregulated, but may be ideally localized to mediate signaling. Collectively, the data above indicate that miR-155-deficient mice exhibit no defect in direct-lytic NK cell tumoricidal function, yet express heightened levels of SHIP-1, which is likely to have consequences on NK functionality.

**Fig 2:**
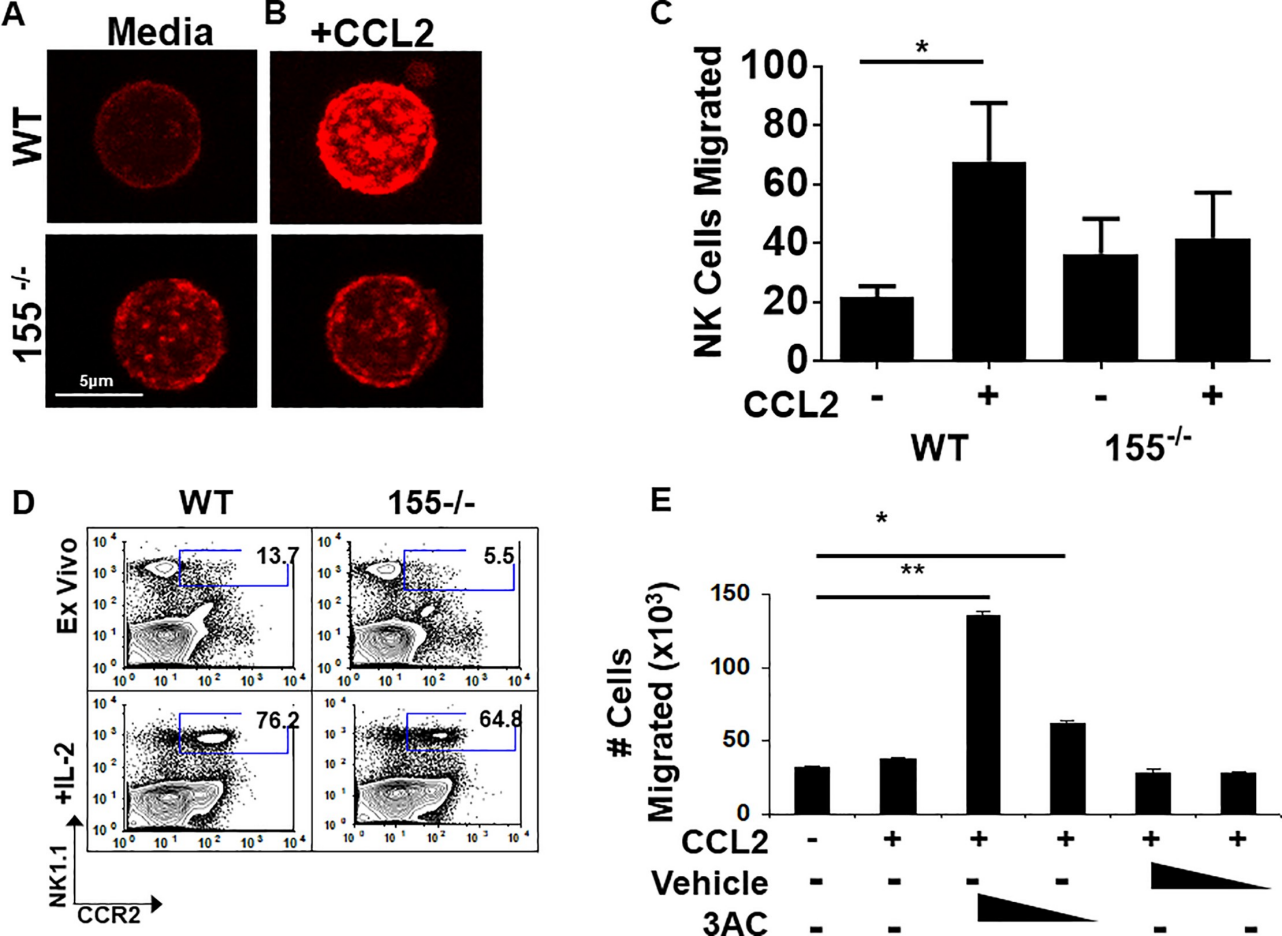
miR-155^-/-^ NK cells exhibit an altered response to CCL2. IL-2 activated NK cells were generated from WT or miR-155^-/-^ purified splenic NK cells. Data shown is a representative of three experiments unless otherwise stated. A) Representative images of untreated or B) CCL-2-treated IL-2 activated cells stained with rhodamine-conjugated Phalloidin to detect F-actin. Magnification = 1833.3x; Scale bar = 2 um. C) A Boyden chamber assay was utilized to detect IL-2 activated cell migration towards a CCL2 stimulus. Data shown is the absolute number of NK cells migrated toward the stimulus as determined by flow cytometry using CountBrite beads. D) Naïve or 24-hour IL-2 activated splenocytes were analyzed by flow cytometry for co-expression of NK1.1 and CCR2. E) Splenocytes were activated with IL-2 for 24h, followed by treatment with the SHIP-1 inhibitor, 3AC. A Boyden chamber assay was then utilized to detect cell migration towards a CCL2 stimulus. Data shown is the absolute number of NK cells migrated toward the stimulus as determined by flow cytometry using CountBrite beads. *P < 0.05, **P < 0.01, ***P < 0.005.

In mammalian cells, SHIP-1 is constitutively associated with filamin-1, a scaffolding protein that organizes polymerized actin filaments into orthogonal angles [20, 21]. Additionally, SHIP-1 negatively regulates phosphoinositide 3-kinase (PI3K) signaling by hydrolyzing phosphatidylinositol 3,4,5-triphosphate (PiP_3_), the second messenger of PI3K that is critical for actin mobilization [28]. SHIP-1-mediated hydrolysis of PiP_3_ results in phosphatidylinositol 4,5-triphosphate (PiP_2_) accumulation. Since PiP_3_ is required for events driving ruffle formation and chemotaxis, loss of this phospholipid subsequently reduces F-actin formation and thus migration [21]. SHIP1 has been found to be involved in all aspects of the NK life cycle [29]. With this abundance of information regarding the relationship between NK cells and SHIP-1, we postulated that the abundance of SHIP-1 could negatively regulate actin polymerization in NK cells. Since polymerization of actin is required for cellular motility, we reasoned that the chemotaxis to tumor-derived factors could be affected by miR-155 deficiency. To address this question, we chose chemokine (C-C motif) ligand 2 (CCL2; also called monocyte chemotactic protein-1 (MCP-1)) as a chemokine stimulus. CCL2 is often overproduced in the tumor microenvironment, including that of breast [30], and attracts T cells, monocytes, and NK cells [10, 31]. Because CCL2 receptor (CCR2) is upregulated on NK cells after activation, we measured responsiveness to CCL2 chemotaxis after stimulation with IL-2 [7]. We compared actin polymerization in CCL2-stimulated, purified NK cells from miR-155-deficient and WT animals by phalloidin staining (which detects filamentous (F)-actin) and subsequent confocal microscopic analysis. In response to CCL2 stimulation, WT NK cells robustly polymerized actin, as visualized by intense F-actin staining in focal pockets on the surface of the cells (Fig 2A). In contrast, CCL2-treated miR-155^-/-^ NK cells did not polymerize actin as compared to media control (Fig 2B). Since F-actin polymerization in response to CCL2 stimulation was defective in those cells lacking miR-155, we hypothesized that miR-155-deficient NK cells may have a reduced capacity to migrate towards a chemokine gradient. To address CCL2 chemoattractive capacity, we measured migration of miR-155-deficient or WT NK cells towards a CCL2 stimulus in a Boyden chamber chemotaxis assay. Using this system, we found that miR-155-deficient NK cells did not migrate towards a CCL2 stimulus, as did WT NK cells (Fig 2C). As a confirmation that the cellular fitness is similar between the NK derived from these mice, a time course assay was performed to evaluate cell viability and marker of NK activation NKp46. The fitness of the WT and miR-155^-/-^ cells were nearly identical. Viability in at all time points under the influence of CCL2 was ~95% for both groups, and NKp46 expression was unchanged at all time points. (S2–S4 Figs). To rule out the possibility that the impaired directional movement was a result of defective CCL2 receptor density, we examined CCR2 expression amongst the strains. In WT mice, at baseline, there are more NK1.1+ CCR2+ NK cells than those of the 155^-/-^ mice (13.7% vs 5.5%; N = 3 mice/strain). However, CCR2 could be induced in both the wildtype and 155^-/-^ NK cells upon stimulation with IL-2, as has been shown previously[7](Fig 2D). Nevertheless, despite comparable receptor expression post-stimulation, miR-155 deficient NK cells remained unresponsive to CCL2, suggesting that the impaired chemotaxis may be a result of dysfunctional actin polymerization. Because SHIP-1 is involved in directional migration and is overexpressed in miR-155^-/-^ NK cells, we sought to determine whether blocking the function of the phosphatase could rescue chemotaxis. We utilized the specific small molecule inhibitor 3-α-aminocholestane (3AC) to block SHIP-1 function [32]. Inhibition of SHIP-1 activity indeed rescued the chemotaxis of miR-155^-/-^ cells to a CCL2 stimulus in a dose-dependent manner (Fig 2E). Collectively, these results suggest that SHIP-1 overexpression resulting from miR-155 deletion confers impaired chemotaxis to CCL2.

### miR-155 deficiency confers impaired NK cell tumor tropism *in vivo*

**Fig 3:**
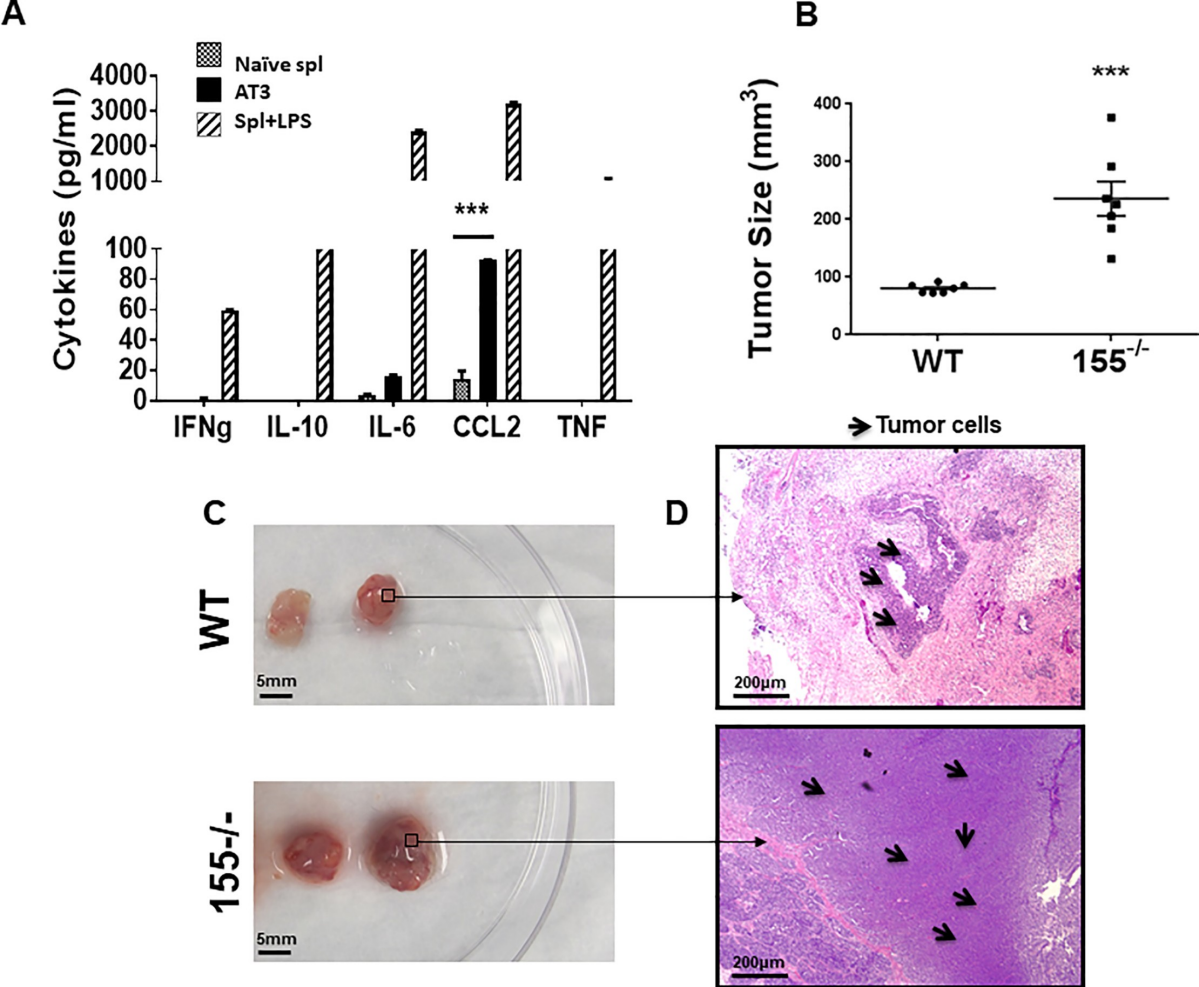
AT3 tumor burden is higher in miR-155^-/-^ hosts. A) Supernatants from AT3 tumor cell cultures (black bars), naïve WT splenocytes (stippled bars), or 24-hour LPS-stimulated splenocytes (grey bars) were collected and analyzed for cytokine production by CBA. B-D) AT3 tumor cells were injected subcutaneously into the flanks of WT or miR-155^-/-^ mice. Four weeks after AT3 implantation, the mice were sacrificed and tumors were measured and dissected from surrounding tissues. B) MiR-155^-/-^ tumor size compared to WT tumor size. C) Two representative AT3 tumors from each mouse strain with D) corresponding hematoxylin and eosin stained representative sections. Magnification = 100x; scale bar = 100 um. *P < 0.05, **P < 0.01, ***P < 0.005.

**Fig 4:**
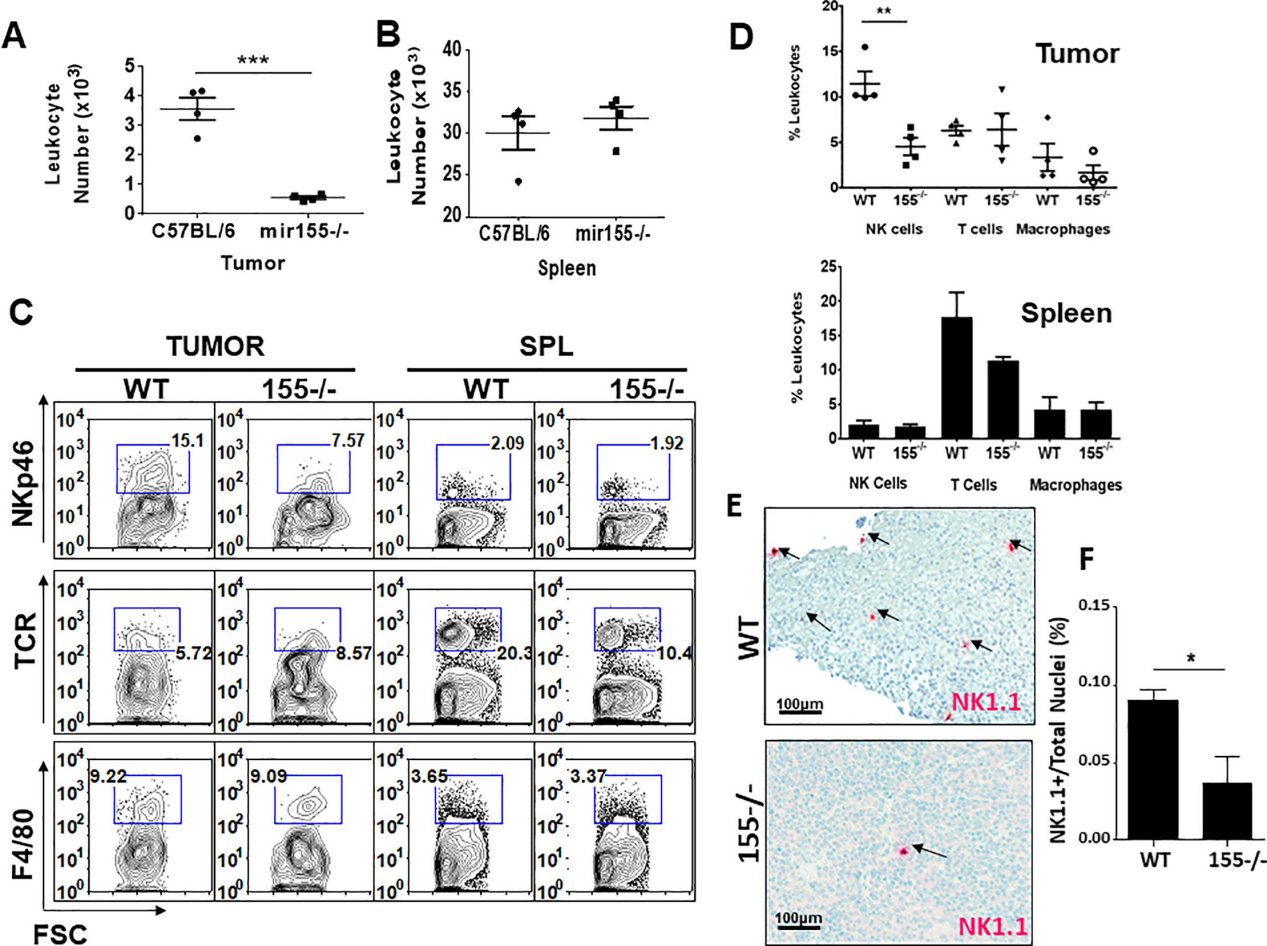
NK cells fail to traffic to AT3 tumors in miR-155^-/-^ hosts. AT3 tumor cells were injected subcutaneously into WT and miR-155^-/-^ mice. Four weeks after AT3 tumor implantation, tumors and spleens were collected and homogenized to single cell suspension for analysis of TILs. Data shown is representative of three experiments. Percentage of NK cells in the tumor A) or spleen B) of tumor bearing mice averaged from 3 experiments. C, D) Representative flow cytometric contour plots and graphs of a single experiment (pooled from 3 mice per strain) showing NKp46+ NK cells in tumor and spleen, as well as TCR+ T cells and F4/80+ Macrophage. E) AT3 tumors from WT or miR-155^-/-^ hosts were fixed, paraffin-embedded and sectioned. The processed tissues were immunostained with anti-NK1.1 to detect intratumoral NK cells, and images of the whole tissues were captured with the Aperio scanning microscope. An imaging algorithm was generated to detect the number of NK1.1+ cells in the tumor. Representative areas of AT3 tumors grafted in WT and miR-155-deficient mice and stained with hematoxylin and anti-NK1.1. Magnification = 200x; Scale bar = 50 um. F) The percentage of NK1.1+ cells in tumors of WT and miR-155-deficient hosts as determined by an imaging algorithm from 2 tumors per strain. The data shown report the mean percentage of NK1.1+ cells of the total nuclei count (as determined by hematoxylin stain) of each entire tumor section. *P < 0.05, **P < 0.01, ***P < 0.005.

Since miR-155-deficient NK cells exhibit impaired chemotaxis to CCL2 ex vivo, we explored the possibility that they would also fail to traffic to a CCL2-rich tumor site. To test the in vivo relevance of miR-155 in NK cell tumor tropism, we first examined whether AT3 mammary carcinoma cells produce chemokines that induce NK homing. We characterized the cytokine and chemokine profile of AT3 cells by using an inflammatory cytokine cytometric bead array (CBA). As positive or negative controls, cytokine production from naïve or lipopolysaccaride (LPS)–stimulated C57BL/6 WT splenocytes was included in the CBA analysis. We found that AT3 produces copious amounts of CCL2 and marginal amounts of IL-6, but not other inflammatory factors such as IFNγ, TNF, or the immunosuppressive cytokine IL-10 (Fig 3A**)**. After confirming their CCL2 production, we examined the in vivo effect of miR-155 deficiency in rejection of AT3 tumors by assessing tumor burden and tumor-infiltrating leukocyte composition. Surprisingly, tumors from miR-155-deficient mice were significantly larger than tumors from WT mice (Fig 3B and 3C). Furthermore, hematoxylin and eosin staining of tumor sections revealed that tumors from miR-155-deficient hosts exhibited a dense lawn of healthy tumor cells (Fig 3D). In contrast, areas of tumor tissue necrosis and infiltrating lymphocytes were present in tumors from WT animals (Fig 3D). We next isolated tumors and analyzed the TIL content by flow cytometry. The total leukocyte number was significantly lower in tumors derived from miR-155^-/-^ background mice **(**Fig 4A**).** The leukocyte number of splenic derived cells however was not significantly different between the two strains **(**Fig 4B**)**. We next evaluated NK cell, T cell and macrophage presence by NK1.1, TCR, and F4/80 staining. Unlike that of T cell and macrophage, NK cells were significantly lower in number in the miR-155 deficient hosts (Tumor-resident NK: miR-155^-/-^ = 4.5% ± 0.9; WT = 11.4% ± 1.3). Splenic NK, T and macrophage numbers were equivalent. ((Splenic NK: miR-155^-/-^ = 1.8% ± 0.3; WT = 2.0% ± 0.5) (Fig 4C and 4D). Our flow cytometric analysis of these NK cells was confirmed with immunohistochemical staining of AT3 tumor sections from WT and miR-155^-/-^ hosts for NK1.1+ NK cells. Immunohistochemistry of AT3 tumor sections from both strains showed noticeably more NK cells infiltrating AT3 tumors grafted in WT hosts (Fig 4E). To quantify data from our immunostained tumor tissues, we scanned tumor sections (N = 2 tumors/strain) with Aperio Scanscope and generated an algorithm in using Definiens TissueStudio Genie software to determine the percentage of NK1.1+ cells per total nuclei in each tumor section. Using this system, we indeed observed significantly fewer NK1.1+ cells in tumor sections of 155^-/-^ hosts (Fig 4F). Collectively, these data suggest that miR-155 deficiency confers impaired NK cell tumor tropism in vivo.

## Discussion

Here, we have shown that miR-155 deficiency in NK cells leads to SHIP-1 overexpression and that this, importantly, leads to faulty chemotaxis in vitro. In vivo, this results in defective tumor tropism and impaired tumor rejection. In this study, our results show that the regulation of tumor infiltration and subsequent NK mediated tumor clearance and responsiveness is regulated by miR-155. With this study, we provide exciting evidence that negative regulation of the actin cytoskeleton via SHIP-1 has consequences in the context of tumor infiltration and cancer rejection. Our results provide novel evidence of a physiological role for SHIP-1 in NK cell tumor infiltration and implicate miR-155 in the regulation of NK cell chemotaxis.

miR-155 expression has a myriad of effects on multiple subsets of the immune system. miR-155 is essential for NK activation, and miR-155 deficiency decreases interferon gamma production. In terms of other subpopulations, miR-155 along with miR-21 have been shown to be involved with MDSC expansion [33], and miR-155 is required for B cell memory response [34]. Additionally, miR-155 deficiency has been shown to enhance MDSC recruitment thus leading to enhancement of solid tumor growth [35]. This previously published work may indicate potential non-NK reasons as to why the tumor burden was higher in the miR-155 mice. In the context of T cells which alongside NK cells are major players in antitumor immunity in the tumor microenvironment, miR-155 has been shown to be overexpressed in effector memory T cells, but not naïve or central memory T cells [36]. In the context of viral infection, T cells have been shown to be defective in the absence of miR-155 [36]. Since T cells have been shown to upregulate miR-155 upon activation, and given the expression of miR-155 in effector memory T cells, miR-155 is thus an essential part of both innate and adaptive immunity. Given that the bic/miR-155^-/-^ mouse used in our studies is not a conditional knockout within NK cells, but rather is a whole-body knockout, the T cell response in vivo may be influencing a substantial amount of the lack of antitumor immunity.

The gene targets of miR-155 in T cells and NK cells are similar. miR-155 in particular has been linked to pattern recognition receptor (PRR) pathways in NK cells, leading to regulation of type I interferon signaling[37]. Similarly, in T cells, miR-155 has been shown to regulate type I interferon signaling, whereby there was increased type I signaling in miR-155 knockout T cells, and defects in proliferation. Type I interferon signaling can affect NK cells equally as it can T cells, leading to defects in STAT signaling, cytokine production, and overall effector function. In terms of Type II Interferons, Banerjee et. al. showed that in T cells, miR-155 skews T cell differentiation to Th1 by inhibiting interferon gamma [38]. Contributing to this, miR-155 overexpression has also been shown to increase T-bet expression [39].

This is particularly relevant in the NK cell context given that interferon gamma production is one of the main ways that NK cells contribute to inflammation and cytotoxicity in the tumor microenvironment. The T cell response is also one that is slower to respond than that of NK. Thus the chromium release assay results may be more representative of a true innate response, while the in vivo results may be more representative of what happens to the immune system as a whole over time. While it is outside of the context of this paper, interrogating the T cell compartment in depth in this model would provide useful–evaluating both T cell memory phenotype and skewing/polarization.

SHIP-1 is involved in the cytoskeletal remodeling of mammalian cells, via regulation of the inositol phospholipids PiP_2_ and PiP_3_. In WT cells, SHIP-1 expression is dampened by induction of miR-155. Since miR-155 depletes levels of SHIP-1, PiP_3_ accumulates at the membrane allowing positive signaling and cellular migration to occur. We found that overexpression of SHIP-1 in NK cells resulting from miR-155 deficiency correlates with decreased motility and abnormal cytoskeletal rearrangement. Thus, miR-155-deficient NK cells may have disrupted motility due to overexpressed SHIP-1 resulting in a lack of PiP_3_ and abundance of PiP_2_ at the membrane. A further analysis of the miR-155-deficient NK cell cytoskeleton is warranted to determine the signaling proteins producing PiP_3_ at the membrane and which actin remodeling proteins are directly associated with SHIP-1 in NK cells. Furthermore, whether SHIP-1 or PiP_3_ are directly involved in actin polymerization in chemotaxing NK cells remains to be determined. Banh et. al. interrogated the effects of SHIP-1 on NK cell development and maturation and found that without SHIP-1, NK cells cannot reach the final stage of development and are functionally immature[40]. In the context of our work, we found higher levels of SHIP-1 expression on miR-155^-/-^ cells, with this SHIP-1 expression rendering the NK cells more dysfunctional. SHIP-1 is important to both development and function of NK cells and plays different roles in this regard. In our study we did not see impaired cytotoxicity in vitro, but significant impairments were seen in in vivo models, whereby the tumor burden was significantly higher and percentage of NK cells in both the tumor and spleen was decreased in the miR-155^-/-^ mouse. It is important in this study to consider effects of the tumor microenvironment, which will be highly suppressive to NK cells. This may either mediated by tumor cell-NK interaction or immune cell-NK interaction (ie. Treg-NK interaction). In a classical cytotoxicity assay, we perhaps did not see reduced cytotoxicity in the miR^-/-^ conditions perhaps due to a lack of contextual cues signaling through SHIP-1 that may indeed be present in a physiologically relevant environment. A limitation of our work is the IL-2 activation of the isolated NK cells performed for all of the in vitro assays. IL-2 may not be the predominant cytokine in a suppressed tumor microenvironment that may be skewed more toward a Th2 or suppressive environment driven by IL-10 and TGFβ [27, 35, 41]. Clearly, further interrogation of miR-155 in the context of tumor induced immune suppression may be warranted.

## Methods

### Mice, cells, and tumor challenge

C57BL/6 and B6.Cg-MiR-155^tm1.1Rsky^/J (*bic/*miR-155^-/-^) mice were purchased from Jackson Laboratory and housed in the Comparative Medicine Facility at the University of South Florida (USF). All experiments were approved by the Institutional Animal Care and Use Committee at USF. Mice were evaluated daily, and humanely euthanized via Carbon Dioxide inhalation. Autoclaved individually ventilated caging (IVC) units (Blueline, Tecniplast, Buguggiate, Italy) were used for all murine housing. A double-sided rack configuration with each rack holding 126 microisolation cages (63 cages per rack side) was used uniformly throughout the murine facility, and each pair of racks was ventilated and exhausted by a single air handling unit (AHU) trolley. Mice were fed an irradiated diet (Envigo, Teklad, Diet 2918) and housed on autoclaved pelleted paper bedding (Envigo, Teklad, 7084). Each cage was provided one nestlet for enrichment (Ancare, Bellmore, NY).

The AT3 mammary carcinoma line was a gift of Dr. Suzanne Ostrand-Rosenberg (University of Maryland, Baltimore, Maryland, United States of America). YAC-1 lymphoma cells were purchased from American Type Culture Collection (ATCC, Virginia, United States of America). AT3 and YAC-1 lymphoma cells were cultured in RPMI supplemented with 10% FBS, 1% Penicillin-Streptomycin, and 1% L-glutamine. For the AT3 tumor challenge, AT3 cells were resuspended to 1x10^7^ c/ml in PBS. Age and sex-matched mice were injected subcutaneously in the right flank with 1 million cells/mouse, and tumors were visually monitored daily for four weeks prior to their sacrifice.

### NK cell isolation

Spleens were harvested from age and sex-matched mice and NK cells were purified with a NK cell negative selection kit (Miltenyi Biotech; 130-090-864). For each isolation, NK cells were determined to be >85% pure by NK1.1/DX5 co-expression. Purified NK cells were cultured in RPMI medium supplemented with 20% FBS, 1% nonessential amino acids, 1% sodium pyruvate, 1% Penicillin-Streptomycin, and 1% L-glutamine (Gibco). For production of activated NK cells, purified NK cells were cultured in the aforementioned media supplemented with 100 U/mL recombinant human IL-2 (Peprotech) for 3 d.

### Flow cytometry

For steady state NK cell analysis, spleens were harvested from age and sex-matched mice, homogenized to single-cell suspension, and immediately stained with NK1.1-APC and DX5-FITC (BD Pharmingen; clones PK136, DX5) and 7-AAD (BD Pharmingen). For receptor phenotyping, 3 d IL-2 activated NK cells were stained with NKp46-FITC, NKG2D-PE, or 2B4-APC (eBioscience; clones 29A1.4, A10, eBio244F4) and 7-AAD. For determination of CCL2 expression, fresh splenic or 24-hour IL-2 [100 U/ml]-stimulated splenic cultures were stained with NK1.1-APC, CCR2-FITC (R&D Systems; FAB5538F) and 7-AAD. In additional experiments, splenic cultures were stained with NK1.1-FITC, NKp46 AF647, TCRB PE, and DAPI was used for dead cell exclusion. For TIL isolation and analysis, spleens and tumors were harvested 4 wk post tumor cell injection. Spleens were homogenized to a single cell suspension and tumors were strained through 70μm strainers to a single cell suspension. The homogenized tissues were stained with NK1.1-APC, DX5-FITC, and 7-AAD. Samples were acquired on a BD FACSCalibur or BD FACSCanto II and analyzed with FlowJo software (BD). All data shown is gated on 7-AAD-negative events falling within the lymphocyte gate (denoted by Forward vs. Side Scatter). For detection of cytokines in tumor supernatants, the Mouse Inflammation Cytometric Bead Assay kit (BD Biosciences; cat#: 552364) was used per manufacturer instructions.

### Chromium release assay

A chromium-51 (^51^Cr)-release assay was performed as described previously [42, 43], using YAC-1 or AT3 tumor cells as targets for IL-2 activated cells or freshly isolated splenocytes. Briefly, target tumor cells were labeled with 200 μCi of Na [^51^Cr] chromate (Amersham Corp., Arlington Heights, IL) in 0.2 ml of medium at 37°C for 1 h. The cells were then washed and added to effector cells at 5 × 10^3^cells/ well in triplicate wells of a 96-well round-bottom plate, resulting in various E/T ratios in a final volume of 0.2 ml per well. After a 5 hour incubation at 37°C, 100 μl of culture supernatants were harvested and counted in with a γ-counter. The percent specific ^51^Cr release was determined as described previously [42, 43] according to the equation: [(experimental cpm − spontaneous cpm)/total cpm incorporated] × 100. All determinations were done in triplicate, and the SEM of all assays was calculated and was typically ~5% of the mean or less.

### Migration assay

Splenocytes from WT or 155^-/-^ mice were incubated for 24 h with IL-2 (100 U/ml) to induce CCR2 surface expression. The cells were then washed and starved in serum and cytokine-free RPMI for 4 hours at 37˚C. For inhibition of SHIP-1, the cells were serum and cytokine starved for three hours and pretreated with 3α-aminocholestane (3AC) (Millipore; 565835) or ethanol vehicle for the final hour of starvation. The cell suspensions were then applied to the top wells of a 10-well Boyden chamber, with CCL2-supplemented [10 ng/ml] RPMI medium in the bottom chamber, separated by a 5-um pore size polyvinylidine fluoride membrane (Whatman). The apparatus was incubated at 37˚C for 2 h. The migrated cells in the bottom wells were then harvested, resuspended to 500 ul, and 50 uL Countbright Absolute Counting Beads (Invitrogen; C36960) were added. For each sample, 20,000 beads were collected. Per manufacturer instruction, the absolute counts of migrated cells were determined by the following equation: Absolute # cells migrated = (# of Cell Events acquired on cytometer / 20,000 beads) x (50,000 beads/50 uL).

### Western blot analysis

Cell lysates were acquired as previously described [43]. Briefly, cells were pelleted and lysed in 1% NP-40, 10 mM Tris, 140 mM NaCl, 0.1 mM PMSF, 10 mM iodoacetamide, 50 mM Na fluoride, 1 mM EDTA, 0.4 mM Na orthovanadate, 10 μg/ml leupeptin, 10 μg/pepstatin, and 10 μg/ml aprotinin, followed by centrifugation to remove nuclei and cell debris. Protein concentrations for each sample were calculated using the Bradford Bio-Rad protein assay. Protein lysates (80 μg/sample) were separated by gel electrophoresis through a 10% SDS-PAGE gel and were transferred overnight at 20 V/hr to a PVDF membrane. The membrane was then probed with anti-SHIP-1 (Santa Cruz Biotechnology; clone P1C1) followed by horseradish peroxidase-conjugated anti-mouse-IgG (GE Healthcare Life Sciences). The blot was developed with a SuperSignal West Femto chemoluminescent reagent (Thermo Scientific). As a protein loading control, the blot was stripped and reprobed for β-actin.

### Immunohistochemistry

Four weeks after tumor implantation, tumors were harvested, bisected, and fixed in 10% formalin. The tissues were then paraffin-embedded, cut into 5 μm sections and mounted onto slides. Representative sections were deparaffinized in two changes of xylene, rehydrated in changes of decreasing ethanol/water solutions, and treated with a citrate-based solution (pH 6.0) for antigen retrieval. The tissue samples were then blocked in 1.5% human serum followed by an avidin/biotin blocking kit (Vector Laboratories), and probed with a biotinylated anti-NK-1.1 (clone PK136, BD Biosciences) overnight at 4°C. Slides were then washed and probed with alkaline phosphatase-conjugated streptavidin (ABC kit; Vectastain) and developed with an avidin/biotin substrate kit (Vector Laboratories). The slides were then counterstained with Gill’s Hematoxylin (Vector Laboratories), dehydrated, and mounted with Cytoseal (Thomas Scientific). The immunostained tumor tissues were scanned with the Spectrum Aperio Scanscope slide scanner, and an algorithm to count immunostained NK1.1+ cells out of total nuclei (detected by hematoxylin) was generated using Definiens TissueStudio software. The percent of tumor infiltrating NK cells was determined with the following equation: ((number NK1.1+ cells/total number of nuclei in tissue)*100). Alternatively, prior to antigen retrieval, some tumors were stained with Gill’s hematoxylin and eosin, dehydrated, and mounted with Cytoseal.

### Confocal microscopy

For F-actin staining, IL-2 activated NK cells were serum and cytokine-starved for 4 h. The cells were then left untreated or treated with 10 ng/ml recombinant mouse CCL2/MCP-1 (R&D Systems) for 30 minutes at 37°C. The cells were fixed in suspension with Fixation buffer (BD Biosciences) and 2.5 x 10^5^ cells were cytospun onto microscope slides (Shandon). The slides were then permeabilized with Permeabilization/Wash buffer (BD Biosciences) and stained with rhodamine-conjugated Phalloidin (Invitrogen) followed by mounting with Fluorescent Mounting Media (Vector Labs). The cells were visualized by confocal microscopy with a 63x oil objective on a Leica TCS SP5 Laser Scanning Confocal Microscope, and captured images were analyzed with LAS-AF software. Definiens Tissue Studio version 4.7 was used to analyze the images for green fluorescence intensity. First a nucleus detection algorithm was applied to the DAPI channel to segment nuclei based on intensity and size thresholds. Next a simple growth algorithm of 1 micron was applied to generate a cytoplasm around each nuclei. The intensity for green fluorescence was measure in each cell, nucleus, and cytoplasm.

### Statistics

Data are presented as means ± standard error of the mean. Differences between individual groups were analyzed by Student’s *t*-test using Graphpad Prism version 7.04. *P* values < 0.05 were considered statistically significant. Significance was also confirmed with the Wilcoxon rank sum test.

Financial Conflict of Interest: Sheng Wei has been a member of the 1000 Talents Program of the People’s Republic of China and a local talents program administered through Tianjin Medical University, but the personal and supporting funding made available through those participations do not appear to be directly related to the work underlying this manuscript. The Wei lab also received unrelated support from Celgene and Amphivena.

## Supporting information

S1 Fig Characterization of miR-155^-/-^ NK cells. Phenotypic and functional analysis of steady state splenic NK cells. Data shown is representative of 3 experiments performed. A) Spleens of WT or miR155^-/-^ mice (N = 3 spleens/strain) were harvested, pooled, and stained with anti-mouse NK1.1 and DX5. B) Percentage of splenic NK cells (NK1.1+/DX5+). C) NK Activating Receptor Phenotype was analyzed by flow cytometry using anti-mouse NKp46, NKG2D and 2B4. D) ^51^Cr-release assay of freshly isolated splenocytes with YAC-1 lymphoma cells and AT3 mammary carcinoma cells as targets.
(TIF)

S2 Fig Time Course Characterization of C57BL/6 (Wildtype) Derived Splenic NK cells. NK cell viability over time (Basal, +30 minutes, +60 minutes, + 120 minutes) under the influence of IL-2 and/or CCL-2. Data shown is representative of 3 experiments performed. Viability was assessed using DAPI.
(TIF)

S3 Fig Time Course Characterization of B6.Cg-MiR-155^tm1.1Rsky^/J (*bic*/miR-155^-/-^) derived Splenic NK cells. NK cell viability over time (Basal, +30 minutes, +60 minutes, + 120 minutes) under the influence of IL-2 and/or CCL-2. Data shown is representative of 3 experiments performed. Viability was assessed using DAPI.
(TIF)

S4 Fig Time Course Characterization of C57BL/6 (Wildtype) and B6.Cg-MiR-155^tm1.1Rsky^/J (*bic*/miR-155^-/-^) derived Splenic NK cells. NKp46 expression was assessed over time (Basal, +30 minutes, +60 minutes, + 120 minutes) under the influence of IL-2 and/or CCL-2. Data shown is representative of 3 experiments performed. Dead cells were excluded via DAPI. Gating scheme is live, singlets that are TCRB-NK1.1+NKp46+.
(TIF)
