# Supplementary figures and images for: MicroRNA-155 governs SHIP-1 expression and localization in NK cells and regulates subsequent infiltration into murine AT3 mammary carcinoma
Source: PLoS One. 2020 Feb 10;15(2):e0225820. doi: 10.1371/journal.pone.0225820 (PMC7010306; doi:10.1371/journal.pone.0225820)

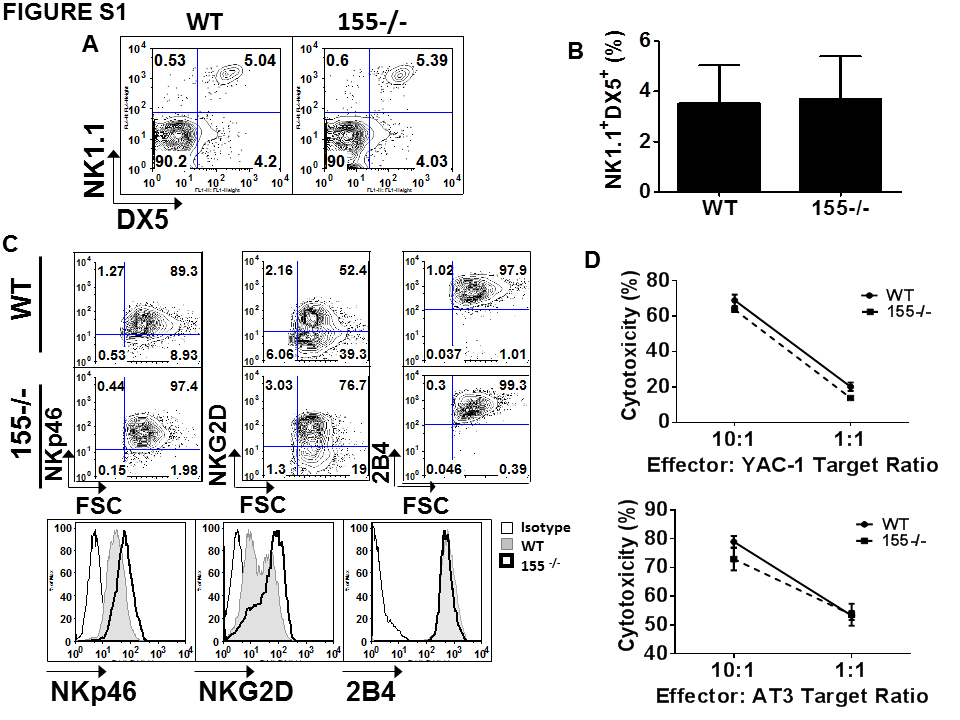

Supplement: S1 Fig — Phenotypic and functional analysis of steady state splenic NK cells. Data shown is representative of 3 experiments performed. A) Spleens of WT or miR155-/- mice (N = 3 spleens/strain) were harvested, pooled, and stained with anti-mouse NK1.1 and DX5. B) Percentage of splenic NK cells (NK1.1+/DX5+). C) NK Activating Receptor Phenotype was analyzed by flow cytometry using anti-mouse NKp46, NKG2D and 2B4. D) 51Cr-release assay of freshly isolated splenocytes with YAC-1 lymphoma cells and AT3 mammary carcinoma cells as targets. (TIF) [file pone.0225820.s001.tif]

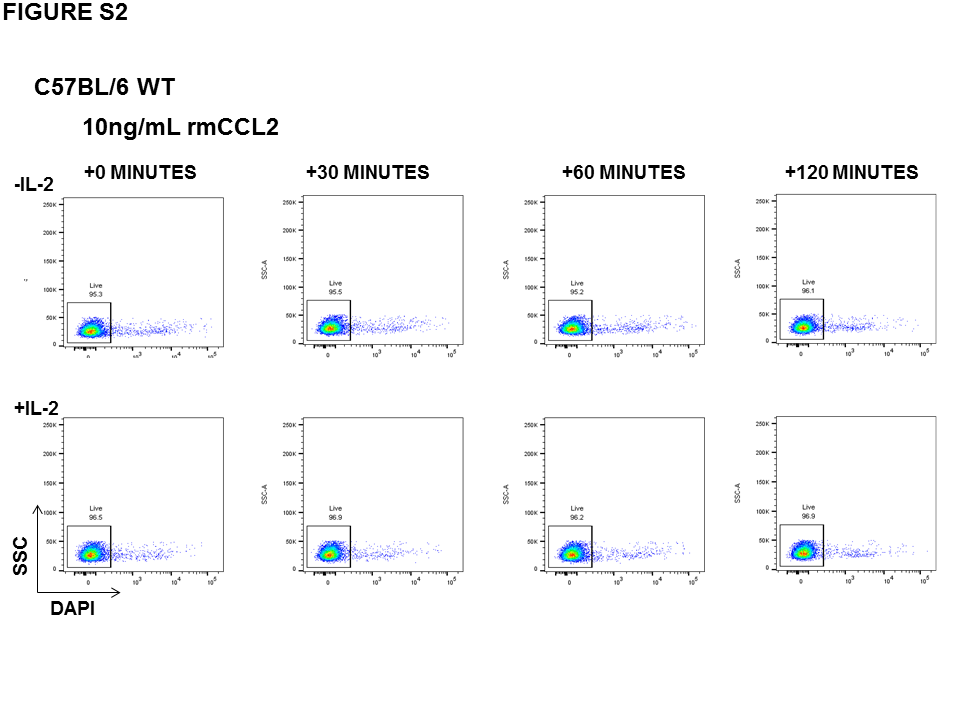

Supplement: S2 Fig — NK cell viability over time (Basal, +30 minutes, +60 minutes, + 120 minutes) under the influence of IL-2 and/or CCL-2. Data shown is representative of 3 experiments performed. Viability was assessed using DAPI. (TIF) [file pone.0225820.s002.tif]

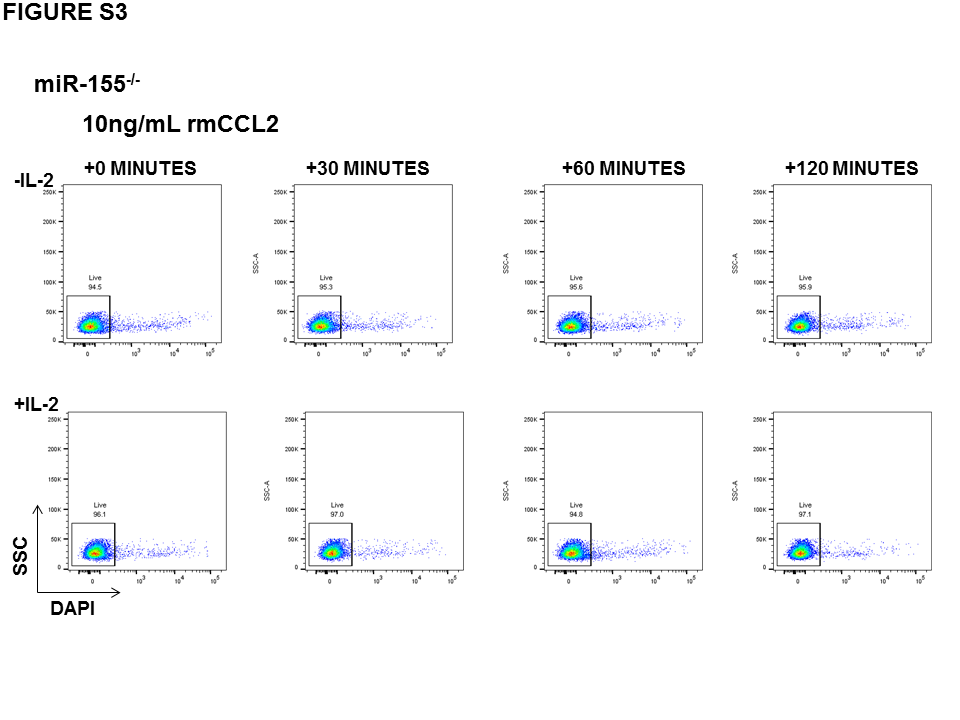

Supplement: S3 Fig — NK cell viability over time (Basal, +30 minutes, +60 minutes, + 120 minutes) under the influence of IL-2 and/or CCL-2. Data shown is representative of 3 experiments performed. Viability was assessed using DAPI. (TIF) [file pone.0225820.s003.tif]

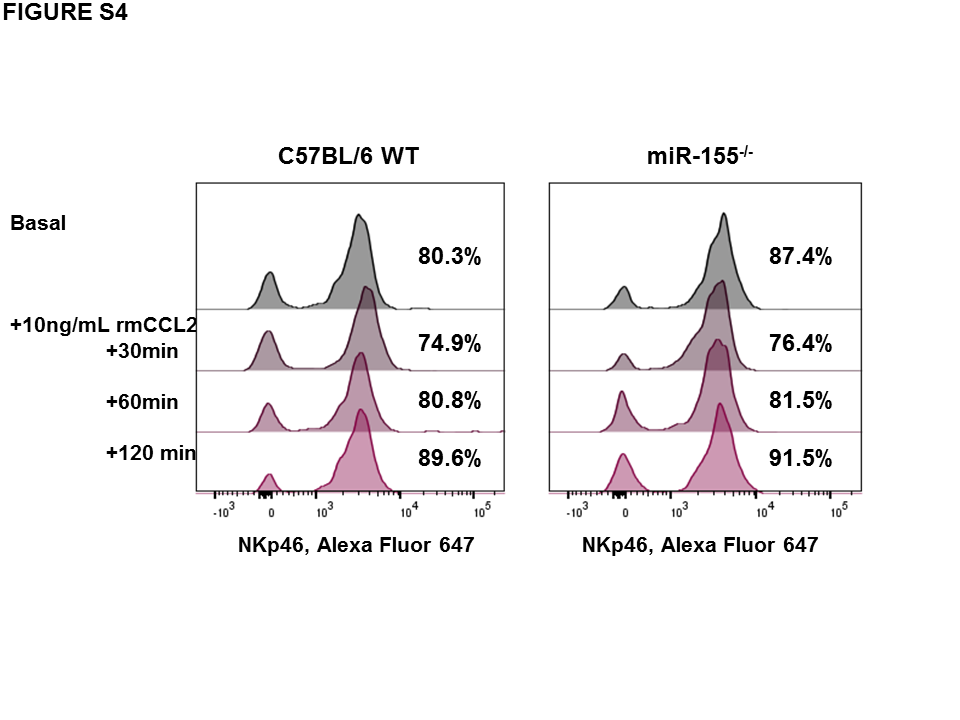

Supplement: S4 Fig — NKp46 expression was assessed over time (Basal, +30 minutes, +60 minutes, + 120 minutes) under the influence of IL-2 and/or CCL-2. Data shown is representative of 3 experiments performed. Dead cells were excluded via DAPI. Gating scheme is live, singlets that are TCRB-NK1.1+NKp46+. (TIF) [file pone.0225820.s004.tif]
